# Is There an Association between Increased Stress and Smartphone Addiction? Insights from a Study on Medical Students from Saudi Arabia during the COVID-19 Pandemic

**DOI:** 10.3390/medicina59081501

**Published:** 2023-08-21

**Authors:** Haytham I. AlSaif, Zeyad A. Alhozaimi, Alhanouf S. Alrashed, Kholoud S. Alanazi, Mohammed G. Alshibani, Turky H. Almigbal, Saad M. Alsaad, Abdullah A. Alrasheed, Fahad D. Alosaimi

**Affiliations:** 1 Department of Family and Community Medicine, College of Medicine, King Saud University, P.O. Box 2925, Riyadh 11461, Saudi Arabia; z.alhozaimi@gmail.com (Z.A.A.); ghatr1416@gmail.com (M.G.A.); talmigbal@ksu.edu.sa (T.H.A.); salsaad@ksu.edu.sa (S.M.A.); aalrasheed1@ksu.edu.sa (A.A.A.); 2 Family Medicine Center, King Saud University Medical City, King Saud University, P.O. Box 7805, Riyadh 11472, Saudi Arabia; 3 Department of Pediatrics, College of Medicine, King Saud University, P.O. Box 2925, Riyadh 11461, Saudi Arabia; alhanoufalrashed622@gmail.com; 4 Clinical Sciences Department, College of Medicine, Vision Colleges, Riyadh 13226, Saudi Arabia; kholoud.s1433@gmail.com; 5 Department of Psychiatry, College of Medicine, King Saud University, P.O. Box 2925, Riyadh 11461, Saudi Arabia; faalosaimi@ksu.edu.sa

**Keywords:** smartphone addiction, depression, psychological stress, academic performance, medical students, Saudi Arabia

## Abstract

*Purpose:* Smartphone addiction is prevalent among medical students, and there is a concern that the coronavirus disease 2019 (COVID-19) pandemic fueled a rise in smartphone addiction. Earlier studies suggest a link between excessive smartphone usage and negative outcomes such as depression, stress, and reduced academic achievement. However, there is a dearth of both local studies in Saudi Arabia and studies conducted during the COVID-19 pandemic exploring the prevalence of smartphone addiction and its association with academic performance, depression, and perceived stress, which is the purpose of the current study. *Methods:* In 2021, a cross-sectional research project took place among medical students at King Saud University and the Vision Colleges located in Riyadh, Saudi Arabia. An online self-administered questionnaire consisting of demographic variables, grade point average (GPA), the Patient Health Questionnaire-9 (PHQ-9), the Perceived Stress Scale-4 (PSS-4), and the Smartphone Addiction Scale—Short Version (SAS-SV) was deployed. *Results:* Three hundred and fifteen students participated. Around 47.9% of students reported smartphone addiction, and the mean SAS-SV score was 32.31 ± 12.01 points. Both PHQ-9 and PSS-4 scores showed a significant positive correlation with the SAS-SV score (r = 0.216, *p* < 0.001 and r = 247, *p* < 0.001, respectively), while GPA did not (r = −0.027, *p* = 0.639). An adjusted analysis showed that the PSS-4 score was positively associated with the SAS-SV score (odds ratio (OR) = 1.206, *p* < 0.001), while the PHQ-9 score was not (OR = 102, *p* = 0.285). *Conclusions:* Smartphone addiction is prevalent among medical students and associated with perceived stress. Additional research is required to gain a deeper comprehension of this issue and to assess the success of intervention initiatives aimed at encouraging healthy smartphone usage, particularly in times of crisis like the COVID-19 pandemic.

## 1. Introduction

A smartphone is a mobile phone with added functionality that includes web browsing, social networking, gaming, and more [1]. Currently, the global count of smartphone users surpasses six billion, and this figure is projected to grow in the coming years [2]. While smartphones offer advantages, they have also been linked to worrisome outcomes, including substantial declines in functionality, emotional distress, withdrawal-like symptoms, cyberspace-oriented relationships, excessive smartphone use, and increased tolerance [3,4,5,6]. Therefore, diagnostic terms related to the aforementioned phenomena, such as “problematic mobile phone use” or “smartphone addiction”, have been suggested in the scientific community [3,4,5]. Smartphone addiction is widespread among medical students. A meta-analysis of research conducted on Asian medical students approximated that around 41.9% experience smartphone addiction [7].

Researchers have become interested in the association between students’ academic performance and their usage of smartphones. An analysis of various studies exploring the link between college students’ smartphone usage and their academic performance revealed that the majority of the research (18 out of 23 studies) identified a negative association, while the remaining studies (5 studies) observed no significant association [8]; indeed, no single study has reported a positive association [8]. Multiple mechanisms were proposed to explain the adverse effects of smartphones on academic performance; for instance, when students use their smartphones, they mostly use them for entertainment at the expense of time allocated for studying [8]. In addition, students may become distracted in class or while studying with task-switching or multi-tasking on their smartphones [8]. This distraction is facilitated by the following: notifications [9], a fear of missing out (FOMO) [10,11], and escaping the sense of boredom associated with academic activities [12]. When mobile phones were banned in English schools, students attained better test results [13].

Smartphone addiction or problematic use has been linked with several mental health issues, including perceived stress and depression. Smartphones and their applications, such as games and social networks, can serve as coping mechanisms for students with mental health issues [5]. Alternatively, excessive smartphone use affects sleep negatively, thus leading to mental health issues [5]. A meta-analysis of studies assessing the link between problematic smartphone use (PSU) and mental health issues in children and young individuals reported increased odds of depression (odds ratio (OR) = 3.17) and perceived stress (OR = 1.86) among those with PSU [14]. Depressive symptoms and perceived stress are common among medical students. Approximately 27.2% of medical students experience depressive symptoms [15]. Medical students suffer from increased levels of perceived stress, according to studies conducted in multiple countries [16,17,18,19].

Severe acute respiratory syndrome coronavirus 2 (SARS-CoV-2) is a respiratory virus discovered in China in 2020 that causes the coronavirus disease 2019 (COVID-19) [20]. In March 2020, the World Health Organization labeled COVID-19 a pandemic due to its rapid transmission across the globe [20]. To curb the transmission of SARS-CoV-2, many countries implemented restrictive measures emphasizing social distancing and working or learning from home [21]. These restrictive measures, in addition to other factors such as anticipatory anxiety and the impact of the illness among family and peers, led to an increase in mental health issues like depression and anxiety, greater use of social media, and more online gaming, which fueled a rise in smartphone addictions [21,22,23].

Research carried out on medical students in Saudi Arabia, specifically in the Western and Qassim regions, indicated a high incidence of smartphone addiction among these students [24,25]. Moreover, almost a quarter of the surveyed Saudi college students felt that smartphone use negatively affected their academic performance [26]. However, there is a dearth of both local studies and studies conducted during the COVID-19 pandemic exploring the association between smartphone addiction and academic performance, depressive symptoms, and perceived stress among medical students. Such local data can inform medical students and advisory bodies at medical colleges about the factors mentioned above that have a significant impact on academic performance and mental health. The objectives of our study were to estimate the prevalence of smartphone addiction and determine the associations between smartphone addiction and academic performance, depression, and perceived stress among medical students in Riyadh, Saudi Arabia. 

## 2. Materials and Methods

### 2.1. Setting and Participants

This was a cross-sectional study targeting medical students enrolled in the College of Medicine at King Saud University (KSU) and the Vision Colleges (VCs). KSU is a public university, while the VCs are private colleges; however, both adhere to a similar curriculum with a preparatory year, which is followed by 5 years of medical college, then an internship year. There were 1466 and 308 medical students enrolled at KSU and the VCs during the 2020–2021 academic year, respectively. In total, there were 702 female students and 1072 male students, of whom 452, 339, 311, 305, and 367 were enrolled in the first, second, third, fourth, and fifth years, respectively. Data were collected during the second semester, from February to March 2021. This study did not include students in preparatory year programs, interns, or students who were not enrolled during the data collection semester. During the time of data collection, lectures were given virtually via online live video streaming, while practical and clinical sessions took place on campus and at affiliated clinical facilities.

### 2.2. Study Instrument

This study utilized a self-administered online questionnaire in English consisting of four parts. The first part consisted of sociodemographic variables that included age, sex, marital status, study year, last semester’s category of grade point average (GPA) (pass, good, very good, or excellent) to measure academic performance, diagnosis of a psychiatric disorder prior to medical school admission, family history of psychiatric disorders, number of smartphone devices owned, the presence of a perceived feeling of increased smartphone usage time during the COVID-19 pandemic, and previous use of a substance with addictive potential (any form of smoking, alcohol, recreational use of prescription drugs, or use of illicit drugs). The second part of the questionnaire included the Smartphone Addiction Scale—Short Version (SAS-SV) [3], which has shown similar validity to those of the full SAS in a sample of college students [27]. The SAS-SV consists of 10 questions, and participants rate their responses on a 6-point scale, ranging from “strongly disagree” (1) to “strongly agree” (6). The overall score falls between 10 and 60 points, with recommended thresholds for identifying smartphone addiction being 31 points for males and 33 points for females [3]. The third section of the survey contained the Patient Health Questionnaire-9 (PHQ-9) for assessing symptoms of depression. The PHQ-9 has previously shown good validity and reliability among medical students [28,29] and measures the occurrence of 9 symptoms of depression in the last 2 weeks using a 4-point Likert scale, where the options are: “not at all” (scored as 0), “several days” (scored as 1), “more than half the days” (scored as 2), and “nearly every day” (scored as 3). The score can range from a minimum of 0 points to a maximum of 27 points. Lastly, the fourth part of the questionnaire included the 4-item Perceived Stress Scale (PSS-4) to assess stress levels; despite being short, this instrument has acceptable validity and reliability [30,31]. To test the accessibility, comprehension, and length of the questionnaire, we conducted a pilot study on a sample of 30 medical students who were not included in this study. A major concern among the respondents was the length of the questionnaire, which was minimized by switching from the 10-item PSS-10 to the 4-item PSS-4.

### 2.3. Data Collection

The minimal sample size required to detect a proportion of around 36.5% of the sample with smartphone addiction with a 5% margin of error and 95% confidence interval (CI) was 299 medical students [24]. We used the convenience sampling technique by obtaining lists of students from student affairs at both colleges. Next, a team of six medical interns reached out to the student batch leaders, distributing the link to the consent form and the online questionnaires via a Google Forms page (Google, LLC, Mountain View, CA, USA) to the electronic messaging groups specific to each batch, which is where course-related updates are typically posted. Two weekly reminders were sent to promote student participation. Students were required to answer all items before being able to submit their questionnaires; therefore, there were no responses with missing items.

### 2.4. Data Analysis

The data gathered for this research were examined using statistical analysis software SPSS version 26.0 (IBM Corporation in Armonk, NY, USA). The internal consistency of the different tools used was tested using Cronbach’s alpha. We utilized descriptive statistics, including measures such as the mean, standard deviation, frequencies, and percentages, to provide a comprehensive overview of both numerical and categorical variables. Bivariate statistical analysis was carried out using appropriate statistical tests (Student’s *t*-test, 1-way analysis of variance (ANOVA), and Pearson’s and Spearman’s correlations) based on the type of study and the outcome variables. A binary logistic regression was employed to examine how certain variables, which showed significant differences in their means, frequencies, or correlation coefficients, impact the likelihood of experiencing smartphone addiction. The statistical significance and precision of the results were reported using a *p*-value of less than 0.05 and 95% confidence interval values.

### 2.5. Ethical Considerations

The Institutional Review Board of the College of Medicine at KSU granted approval for this study (project nos. E-20-4609 (KSU) and E-20-4620 (VCs)). Permission to use SAS-SV was obtained from Dr. Kwon [3], while permission was not required to use PHQ-9 and PSS-4 for research purposes. Informed consent was provided at the top of the questionnaire page for viewing before participating voluntarily. No incentives were offered for participation. The information given covered this study’s objective, the promise of confidentiality for participants’ responses, and the contact details for any further inquiries. Personal identification details, such as full name, student identification number, contact number, and email, were not gathered. The data were kept on the computer of the principal investigator, and this computer was secured with a password.

## 3. Results

### 3.1. Participant Characteristics

A total of 315 medical students responded (response rate = 17.8%), which satisfies the power requirement based on the sample size calculation. The response rate was higher among VC students (52.9%) compared to KSU students (10.4%) and higher among female students (24.9%) than male students (13.1%). Furthermore, the highest response rate was 23.7% among fifth-year students, followed by 19.7% among first-year students, while response rates were almost similar between second-year students (15.9%) and third- and fourth-year students (both 13.8%). Overall, there were more female respondents (55.6%), and the majority (92.7%) of participants were single. The lowest proportions of participants were from the fourth (13.3%) and third (13.7%) years. Less than half (44.8%) of the respondents had a very good GPA. Only a small proportion of participants had been diagnosed with a psychiatric disease prior to medical school admission (11.1%) or had a family member with a psychiatric illness (21%). Half (49.5%) of the participants had just one smartphone device, and 2/3 (67%) of the participants felt that their smartphone usage had increased during the COVID-19 pandemic. Lastly, 1/5 (21%) of the participants reported previous use of a substance with addictive potential, with any form of smoking being the most commonly reported type of substance use among the participants (*n* = 63, 95.5%). Table 1 shows the sociodemographic characteristics of the study participants.

### 3.2. SAS-SV

The Cronbach’s alpha value for the SAS-SV was 0.898, indicating good internal consistency. The median SAS-SV score was 31 (interquartile range (IQR) = 22–42) points. Significantly higher mean SAS-SV scores were observed in the following groups: females, single individuals, students who were not diagnosed with a psychiatric illness prior to medical school admission, students who felt that their smartphone use had increased during the COVID-19 pandemic, and students who had not previously used a substance with addictive potential. There were no significant differences between the mean SAS-SV scores for different GPA categories. Table 1 presents the mean SAS-SV scores across categories of sociodemographic characteristics. Almost half of the participants (47.9%) scored above the threshold for addiction. A slightly greater frequency of addiction was observed among female students than among male students (χ^2^ = 0.499, *p* = 0.48). Table 2 shows the frequency of smartphone addiction based on the participating students’ sex. 

The highest mean scores for the SAS-SV questions were for “using my smartphone longer than I intended” (3.79 ± 1.68 points), followed by “having a hard time concentrating in class, while doing assignments, or while working due to smartphone use” (3.62 ± 1.75 points). On the other hand, the lowest mean scores were recorded for “the people around me tell me I use my smartphone so much” (2.87 ± 1.64 points), followed by “having my smartphone in my mind even when I am not using it” (2.89 ± 1.61 points). Table 3 shows the students’ responses and the mean score for each of the SAS-SV items.

### 3.3. PHQ-9 and PSS-4

The Cronbach’s alpha value for the PHQ-9 was 0.904, indicating excellent internal consistency, and the median PHQ-9 score was 10 (IQR = 5–15) points. The mean PHQ-9 scores were significantly higher in the following groups: females, single individuals, students who felt that their smartphone use had increased during the COVID-19 pandemic, and students who had never previously used a substance with addictive potential. The Cronbach’s alpha value for the PSS-4 was 0.722, indicating acceptable internal consistency, and the median PSS-4 score was 8 (IQR = 5–10) points. The mean PSS-4 scores were significantly higher across the following groups: fifth-year students, students who only owned one smartphone device, and students who felt that their smartphone use had increased during the COVID-19 pandemic. Table 1 shows the mean PHQ-9 and PSS-4 scores across categories of sociodemographic characteristics.

### 3.4. Correlation between SAS-SV and Other Measures

There was a small positive correlation between smartphone addiction and perceived stress (r = 0.247, *p* < 0.001) and depressive symptoms (r = 0.216, *p* < 0.001). Table 4 shows the correlation coefficients between smartphone addiction and depressive symptoms, perceived stress, and academic performance. 

### 3.5. Likelihood of Smartphone Addiction

The binary logistic regression model showed that students who felt that their smartphone use had increased during the COVID-19 pandemic had the greatest likelihood of being addicted to their smartphones (odds ratio (OR) = 3.4); moreover, for each 1-point increase in the PSS-4 score, the likelihood of smartphone addiction increased by 1.2 times (OR = 1.21). On the other hand, students with prior use of substances with addictive potential had a reduced likelihood of smartphone addiction (OR = 0.45). Table 5 shows the results of the binary logistic regression model.

## 4. Discussion

In our research, we found that smartphone addiction was observed in 47.9% of the participants. This rate was greater than the prevalence noted among final-year medical students in the Western region of Saudi Arabia, which stood at 36.5 [24]. Nevertheless, it remained lower compared to the percentage reported among medical students in the Qassim region of Saudi Arabia, which was 60.3% [25]. In our study, we noticed that the mean SAS-SV scores were lower among junior medical students (first-, second-, and third-year students); however, the difference in mean scores between the academic years was not statistically significant. This might explain why the prevalence was higher than that reported by Alhazmi et al., who only included final-year medical students, and lower than that reported in the study by Alsalameh et al., in which junior medical students composed more than 2/3 of the total sample [24,25]. Our reported prevalence is slightly higher than the prevalence among Asian medical students (41.9%; 95% CI, 36.2–47.7) [7]. 

We determined that stress was associated with smartphone addiction even after controlling for other variables, including depressive symptoms. Stress can lead to increased smartphone use through multiple mechanisms. First, increased stress levels lead to a reduction in self-control, which is followed by an excessive use of smartphones [32]. Second, excessive smartphone usage for hedonic purposes (entertainment, social media, and games) might be an escape mechanism from perceived stress [33,34]. Third, the relationship between perceived stress and PSU among Chinese university students was influenced by the mediation of FOMO [35]. Students attempt to relieve their stress by constantly checking their smartphones for messages, following what their peers are doing and what is trending on social media [35,36]. A weak positive correlation between smartphone addiction and depressive symptoms was observed in the current study. However, the adjusted analysis did not show an association. There are factors that might limit the use of smartphones among depressed people; for instance, depression leads to social isolation, resulting in less use of smartphones for communication [37]. Alternatively, other factors might increase smartphone usage among depressed individuals. For instance, constantly checking the phone might be a sign of seeking social reassurance from other people, reflecting low self-esteem, which is one of the depressive symptoms [5].

In the study by Alosaimi et al. on Saudi college students, students felt that their use of smartphones negatively affected their academic performance [26]. Nevertheless, upon gathering their self-reported GPAs, we found no significant variation in smartphone addiction scores among different GPA groups. This aligns with a smaller portion of research in the field, while the majority indicated a negative association [8]. Interestingly, despite not finding an association between GPA and smartphone addiction, the SAS-SV questions most students agreed with during our study were related to smartphones being used longer than intended and their negative impact on concentration during academic activities. Both of these inquiries are part of the suggested explanations for how smartphone addiction negatively impacts academic performance [8]. 

In the current research, we examined the use of substances with addictive potential, and smoking was the most commonly reported substance used by students. We found a negative association between smartphone addiction and the use of substances with addictive potential. While examining the available literature, most research conducted on young individuals (both university and medical students) discovered no correlation [24,38,39,40,41]. On the other hand, Liu et al. observed a positive association among Chinese medical students, and Dey et al. observed a negative association among Swiss young men [42,43]. A possible explanation for this negative association is that students might substitute smoking for smartphone use [42]. Qualitative studies could be useful to further explore the link between smartphone addiction and other habits with addictive potential, such as smoking, from the perspectives of students or young people.

Two-thirds of the students in our study perceived that their smartphone use had increased during the COVID-19 pandemic, and this association remained even after adjusting for other factors in the regression analysis. Similarly, Jordanian university students (including medical students) reported increased smartphone use during the COVID-19 quarantine [44]. Furthermore, a study from Bangladesh conducted during the COVID-19 pandemic among high school and university students reported an increased prevalence of smartphone use compared to a study of a similar population conducted before the pandemic [45,46]. However, we did not notice a similar pattern, as pre-pandemic studies among Saudi medical students reported both lower (36.5%) and higher (60.3%) estimates than ours (47.9%) [24,25].

### Strengths and Limitations

This research was conducted during the COVID-19 pandemic and aimed to examine the extent of smartphone addiction, its association with academic achievements, depressive symptoms, and perceived stress among Saudi students from both private and public medical colleges. However, there are multiple limitations to this study, including its reliance on the self-reported data of the participants and its use of the convenience sampling technique. This study was a cross-sectional study; therefore, we cannot be certain about the direction of the associations found. The sample size was suitable for estimating the prevalence of smartphone addiction; however, a larger sample size would have enabled more precise logistic regression results. Other relevant variables related to smartphone addiction, such as the methods of internet access on smartphones and current psychiatric comorbidities, were not collected.

## 5. Conclusions

The extent of smartphone addiction among Saudi medical students during the COVID-19 pandemic was relatively high. Furthermore, after making adjustments in the analysis, a positive association was found between excessive smartphone usage and the perception of stress. Moreover, the consumption of addictive substances was determined to have a negative relationship with smartphone addiction. Nevertheless, we found no association between smartphone addiction, depressive symptoms, and academic performance. We recommend that medical students and advisory bodies in medical colleges be mindful of and proactively address the harmful interaction between smartphone addiction and perceived stress on student mental health and well-being, especially in times of crisis similar to the COVID-19 pandemic. Finally, it could be beneficial to conduct qualitative and prospective longitudinal research to investigate how smartphone addiction, academic achievement, depressive symptoms, perceived stress, and substance usage, particularly smoking, intersect among medical students.

## Figures and Tables

**Table 1:** Distribution of sociodemographic characteristics of study participants (*n* = 315).

Sociodemographic Characteristic		Number of Students (%)	Mean SAS-SV Scores ± SD, *p*-Value	Mean PHQ-9 Scores ± SD, *p*-Value	Mean PSS-4 Scores ± SD, *p*-Value
Type of college	Private	163 (51.7)	32.01 ± 12.58	10.49 ± 6.54	7.12 ± 3.41
	Public	152 (48.3)	32.63 ± 11.39	9.80 ± 7.31	6.93 ± 3.20
			*p* = 0.645	*p* = 0.379	*p* = 0.615
Sex	Male	140 (44.4)	30.80 ± 12.30	9.22 ± 6.69	7.00 ± 3.13
	Female	175 (55.6)	33.52 ± 11.6	10.90 ± 7.03	7.05 ± 3.46
			*p* = 0.046	*p* = 0.033	*p* = 0.906
Marital status	Single	292 (92.7)	32.67 ± 11.92	10.24 ± 6.85	7.07 ± 3.29
	Married	19 (6)	23.89 ± 9.43	6.78 ± 6.16	6.15 ± 3.86
	Divorced, separated, or widowed	4 (1.3)	46.25 ± 6.65	19.75 ± 6.34	8.25 ± 0.50
			*p* < 0.001	*p* = 0.002	*p* = 0.387
Study year	First	89 (28.3)	34.73 ± 12.87	10.98 ± 7.18	6.86 ± 3.82
	Second	54 (17.1)	32.35 ± 12.23	10.64 ± 5.73	7.29 ± 3.49
	Third	43 (13.7)	31.13 ± 11.97	10.27 ± 7.72	5.62 ± 2.96
	Fourth	42 (13.3)	30.14 ± 10.93	8.71 ± 6.75	7.12 ± 2.56
	Fifth	87 (27.6)	31.44 ± 11.31	9.64 ± 6.99	7.68 ± 2.95
			*p* = 0.212	*p* = 0.425	*p* = 0.018
GPA	Excellent	121 (38.4)	33.29 ± 12.29	9.56 ± 6.34	7.04 ± 3.47
	Very good	141 (44.8)	30.92 ± 11.51	10.13 ± 7.13	6.90 ± 3.19
	Good	47 (14.9)	34.67 ± 12.37	11.42 ± 7.17	7.40 ± 3.41
	Pass	6 (1.9)	26.00 ± 11.41	13.00 ± 10.77	7.00 ± 2.68
			*p* = 0.096	*p* = 0.324	*p* = 0.847
Diagnosed with a psychiatric disorder prior to medical school admission	Yes	35 (11.1)	27.22 ± 13.69	11.85 ± 5.88	6.25 ± 3.39
	No	280 (88.9)	32.95 ± 11.65	9.94 ± 7.02	7.12 ± 3.29
			*p* = 0.008	*p* = 0.124	*p* = 0.143
Family history of psychiatric disorders	Yes	66 (21)	30.87 ± 11.41	10.21 ± 6.97	7.34 ± 2.85
	No	249 (79)	32.69 ± 12.15	10.14 ± 6.92	6.94 ± 3.42
			*p* = 0.275	*p* = 0.944	*p* = 0.383
Number of smartphone devices owned	1	156 (49.5)	32.86 ± 11.06	10.70 ± 7.34	8.03 ± 2.74
	2	99 (31.4)	31.81 ± 12.29	9.69 ± 6.35	6.16 ± 3.37
	3 or more	60 (19.1)	31.70 ± 13.90	9.50 ± 9.69	5.85 ± 3.79
			*p* = 0.722	*p* = 0.377	*p* < 0.001
Perceived feeling of increased smartphone usage time during the COVID-19 pandemic	Yes	211 (67)	34.72 ± 11.85	11.35 ± 7.08	7.82 ± 3.08
	No	104 (33)	27.43 ± 10.85	7.74 ± 5.90	5.41 ± 3.17
			*p* < 0.001	*p* < 0.001	*p* < 0.001
Have you ever used a substance with addictive potential?	Yes	66 (21)	29.62 ± 12.35	12.12 ± 7.14	7.65 ± 3.23
	No	249 (79)	33.02 ± 11.84	9.63 ± 6.78	6.86 ± 3.32
			*p* = 0.040	*p* = 0.009	*p* = 0.087
Total		315	32.31 ± 12.01	10.16 ± 6.92	7.03 ± 3.31

GPA, grade point average; PHQ-9, Patient Health Questionnaire-9; PSS-4, 4-item Perceived Stress Scale; SAS-SV, Smartphone Addiction Scale—Short Version; SD, standard deviation.

**Table 2:** Frequency of smartphone addiction based on participating students’ sex (*n* = 315).

	Frequency (%)		Total
	Male	Female	*p* Value * = 0.48
Addicted	64 (45.7)	87 (49.7)	151 (47.9)
Not addicted	76 (54.3)	88 (50.3)	164 (52.1)
Total	140	175	315

* Chi-squared test for association: χ^2^(1) = 0.499.

**Table 3:** Students’ responses and the mean score for each of the Smartphone Addiction Scale—Short Version (*n* = 315) question.

SAS-SV Question	Mean ± SD
Missing planned work due to smartphone use	3.36 ± 1.6
2. Having a hard time concentrating in class, while doing assignments, or while working due to smartphone use	3.62 ± 1.75
3. Feeling pain in the wrists or at the back of the neck while using a smartphone	3.01 ± 1.72
4. Won’t be able to stand not having a smartphone	3.51 ± 1.72
5. Feeling impatient and fretful when I am not holding my smartphone	2.92 ± 1.59
6. Having my smartphone in my mind even when I am not using it	2.89 ± 1.61
7. I will never give up using my smartphone even when my daily life is already greatly affected by it	3 ± 1.65
8. Constantly checking my smartphone so as not to miss conversations between other people on Twitter or Facebook	3.29 ± 1.64
9. Using my smartphone longer than I had intended	3.79 ± 1.68
10. The people around me tell me that I use my smartphone too much	2.87 ± 1.64

**Table 4:** Correlation of smartphone addiction with other variables (*n* = 315).

Variable	Smartphone Addiction Scale—Short Version (SAS-SV)		Current Cumulative GPA Category *		Depressive Symptoms (PHQ-9) **		Perceived Stress (PSS-4) **	
	Correlation Coefficient (r)	*p*-Value (*p*)	r	*p*	r	*p*	r	*p*
SAS-SV	1	0	−0.027	0.639	0.216	<0.001	0.247	<0.001
GPA	−0.027	0.639	1	0	−0.107	0.057	−0.061	0.283
PHQ-9	0.216	<0.001	−0.107	0.057	1	0	0.273	<0.001
PSS-4	0.247	<0.001	−0.061	0.283	0.273	<0.001	1	0

* Spearman’s correlation; ** Pearson’s correlation.

**Table 5:** Binary logistic regression for the association between sex, marital status, depressive symptoms, stress, and use of a substance with addictive potential and smartphone addiction (SAS-SV) *.

	B	S.E.	Wald	*p*-Value	OR	95% CI for OR	
						Lower	Upper
Constant	−2.136	0.670	10.152	0.001	0.118		
Sex	−0.110	0.258	0.183	0.669	0.896	0.540	1.485
Marital status	0.062	0.389	0.026	0.873	1.064	0.496	2.283
Diagnosed with a psychiatric disorder prior to medical school admission	−0.818	0.427	3.672	0.055	0.441	0.191	1.019
Perceived feeling of increased smartphone usage time during the COVID-19 pandemic	1.223	0.292	17.517	<0.001	3.398	1.916	6.026
Have you ever used a substance with addictive potential?	−0.790	0.331	5.701	0.017	0.454	0.237	0.868
PHQ-9	0.022	0.020	1.145	0.285	1.022	0.982	1.064
PSS-4	0.187	0.047	16.188	<0.001	1.206	1.101	1.321

B, unstandardized regression coefficient; S.E., standard error; OR, odds ratio; CI, confidence interval. * Nagelkerke R^2^ = 0.257.

## Data Availability

The original contributions presented in this study are included in the article; further inquiries can be directed to the corresponding author.
